# History of a Case of Palsy, Cured by Lightning. Also, Minutes of a Case of Yaws: In a Letter from Dr. Thomas Humphreys, of Lynchburgh, Virginia, to Dr. Benjamin Rush, Dated November 1st, 1803

**Published:** 1808-02

**Authors:** 


### *History of a Case of Palsy, cured by Lightning. Also, Minutes of a Case of Yaws: in a Letter from* Dr. Thomas Humphreys, *of Lynchburgh, Virginia, to* Dr. Benjamin Rush, *dated November 1st,* 1803.

Mrs. Susannah Wiatt, of the state of Virginia, and county of King and Queen, a lady of great respectability, family, and fortune, informed me, that about the 49th year year of her age, while on a visit at some distance from home, a considerable fall of snow took place, followed with hail and sleet, accompanied with ice; next day the weather being somewhat settled, on her return home, her carriage broke down, which occasioned a walk of near ten miles, and took her part of two days to accomplish: on her arrival at home, she found herself in a profuse sweat, but next day felt chilly all over, nor were her friends able, from every possible exertion, to restore her natural warmth; she felt herself in a very great deal of misery, and notwithstanding medical aid being called in, and her health partly reinstated, yet her pains in a short time became exceedingly severe, and in less than a year, she partly lost the use of both her arms.

An eminent physician, Dr. John Brockenbury, of Essex county, Virginia, being called to attend her, among other parts of his treatment, electrified her once a fortnight, and sometimes twice in a week, for two years, without any other apparent advantage whatsoever, than that of deriving a mere temporary relief. Three years having now expired; in the course of the fore part of the summer, she was more frequently electrified than before. Some time in the month of July, about 3 P. M. being in a lower room in the dwelling-house, one of her sons and two negro servants being also present, (the atmosphere at that time being heavily charged with electric matter) just as she got up from the dinner table, and had reached the side of a bed that stood in the said room, a sudden and violent explosion took place. She was instantaneously struck down, and fell motionless across the bed.

The bushes that were in the chimney were burnt, and several dead birds were found there; all the plastering in the inside of the chimney was stripped off, reduced to a very soft consistency, and was spattered all over the room: so hot was this soft mortar, that whatever part of the negroes skins it was dashed against, was immediately blistered. There was also a large round ball, formed of this soft or fused mortar, which rolled along the floor of the room until it came to a cat-hole in the door, and being larger than the hole, it remained there apparently red hot. The young gentleman touching it with his finger, was severely burnt; on pushing a stick into it, in a few minutes its action on the enclosed wood exhibited demonstrative proof of the intensity of its heat. In the middle of the ceiling of the room, was a large black circle or ring, with a white spot exactly in the centre. Query, What was this owing to?

to? was it owing to the back-stroke or reaction of the electric fluid? no injury being done to any of the inside plastering, either on the ceiling or walls. And again, upon what philosophical principle can the instantaneous fusion of this intensely exsiccated mortar be accounted for? But to return to the lady.

The young gentleman, her son, and the two servants, who were none of them otherwise hurt than by the burning plastering as already mentioned, after recovering from the surprise occasioned by the violent and dreadful report, they, with great presence of mind, loosened her clothes, ripped open her stays, and, by sprinkling water on her, shaking and pulling her about, and using constant friction, after some short time, recovered her: she demanded to know what they were doing, and what occasioned all the confusion apparently about her, and received for answer— that the house had been struck with lightning, and that the first they observed of her, she lay across the bed to every appearance dead. As soon as she was sufficiently recovered to walk, she went up stairs, to examine whether the house had not taken fire in any of the upper apartments; on coming down stairs she made a false step, and instantly stretching out her arm, caught hold of the hand-railing of the staircase, and exclaimed aloud to her son, to come and behold how miraculously she had recovered the use of her hands, and to observe with what ease she could extend her arms, and grasp, at pleasure, the bannistering of the staircase.

Next day the physician coming to electrify her as usual, was pleased and astonished, as well as agreeably surprised, to find that his patient had experienced that complete relief, from a moment's application of atmospheric electricity, administered by nature's omnipotent arm, which two years unwearied application of artificial electricity was not able to effect.

None of the other people in the room being struck but the lady, the physician supposed her system was, (in consequence of the many and repeated electrifications she had undergone, and particularly of late,) rendered much more susceptible of the electric fluid, than any of the others in the room. It is now about twenty years since that time, and the lady informed me, she never has had the slightest touch of the rheumatism since she received the said shock. She now enjoys good health.

To

To Dr. Humphreys.

Sir,

In the year 1770, about the month of June, I had a number of African slaves for sale; among them was a lad about eighteen years of age, who was a miserable object, from the disorder called the yaws; he was vastly more afflicted with it than any person I ever saw before or since; from his head to his feet he was thickly set with those sorts of knots or ulcers, which that disorder produces when it is in its worst stages. A Doctor Ellis lived at the place, who had very deservedly acquired great reputation for his professional merit. I applied repeatedly to him to cure this lad: he did not actually refuse, but signified it would be tedious, the cure doubtful, and probably more expensive than he was worth. In this situation I should not have hesitated to have given him away to any one that would have accepted of him. Among my slaves there were a few who had been living for some time at one of the British factories in Africa, and understood a little of the English language. Observing that I was at a loss what to do with this diseased slave, they undertook to cure him, to which I readily consented, but with little faith in their success.

*The Cure was as follows.*

They took him to a running stream of water, laid him in it, two confined his feet, two his arms, and one held up his head to prevent drowning; two then operated in scrubbing off the knots and ulcers in the running water. The operation must have been dreadful, for they scrubbed him with corn-husks, and even sand; the blood and matter and scabs were constantly washed down the stream: when every ulcer was thus smoothed away and cleansed by the running water, they led him up naked to their house, and wiped him; then they made an ointment of the juice of limes made boiling hot, and mixed to a proper consistence, with powdered iron scales taken from a blacksmith's anvil: with this ointment they anointed every sore with a feather; the same operation was continued for four weeks; every six or seven days, they gave him frequently a decoction of some roots, which, I believe, operated as a purgative; in about eight weeks they completed a cure; in three or four months he became sleek, fat, and a very likely fellow; all the sores skinned over, and no scar remained. I sold him afterwards, and never heard that the complaint returned. Doctor Ellis was not less astonished at the cure than I was; he could not account for it. He supposed the iron and lime juice formed a species of salts, with which he he was unacquainted. He promised to give the subject full consideration, and to inform me of the result; but he died soon afterwards, and I found nothing among his papers, but a state of the case similar to the above.

DAVID ROSS.

